# Haste Makes Waste: Accelerated Molt Adversely Affects the Expression of Melanin-Based and Depigmented Plumage Ornaments in House Sparrows

**DOI:** 10.1371/journal.pone.0014215

**Published:** 2010-12-03

**Authors:** Csongor I. Vágási, Péter L. Pap, Zoltán Barta

**Affiliations:** 1 Behavioural Ecology Research Group, Department of Evolutionary Zoology, University of Debrecen, Debrecen, Hungary; 2 Department of Taxonomy and Ecology, Babeş-Bolyai University, Cluj Napoca, Romania; University of Western Ontario, Canada

## Abstract

**Background:**

Many animals display colorful signals in their integument which convey information about the quality of their bearer. Theoretically, these ornaments incur differential production and/or maintenance costs that enforce their honesty. However, the proximate mechanisms of production costs are poorly understood and contentious in cases of non-carotenoid-based plumage ornaments like the melanin-based badge and depigmented white wing-bar in house sparrows *Passer domesticus*. Costly life-history events are adaptively separated in time, thus, when reproduction is extended, the time available for molt is curtailed and, in turn, molt rate is accelerated.

**Methodology/Principal Findings:**

We experimentally accelerated the molt rate by shortening the photoperiod in order to test whether this environmental constraint is mirrored in the expression of plumage ornaments. Sparrows which had undergone an accelerated molt developed smaller badges and less bright wing-bars compared to conspecifics that molted at a natural rate being held at natural-like photoperiod. There was no difference in the brightness of the badge or the size of the wing-bar.

**Conclusions/Significance:**

These results indicate that the time available for molt and thus the rate at which molt occurs may constrain the expression of melanin-based and depigmented plumage advertisements. This mechanism may lead to the evolution of honest signaling if the onset of molt is condition-dependent through the timing of and/or trade-off between breeding and molt.

## Introduction

The coloration of animal integument, such as skin, scales, fur and feathers, is often determined by deposits of pigments. Melanins are the most common pigments present in almost all birds and can be categorized into two types: phaeomelanins are for various shades of yellowish and rufous brown, while eumelanins for grey, brown and black [1], [2]. In addition to their ubiquitous role in naturally selected functions like crypsis, protection against solar UV-radiation and mechanical fatigue, melanin-based coloration also frequently serves as sexual or social signal [3]. Beyond pigmentary colors, several avian species possess feather patches that lack pigments, the so-called depigmented white ornaments, which also act as signals used in intra- and intersexual communication (e.g. the forehead patch in male collared flycatchers *Ficedula albicollis* [4], [5]). These ornaments are built of finely structured, nanometer-scale matrix of keratin and variably-sized air vacuoles with or without spongy layer and are perceived as white because they incoherently scatter the whole spectrum of incident light [6].

A central tenet of evolutionary signaling theory posits that a communication system where information is conveyed via ornaments is susceptible to cheaters' invasion unless a significant and quality-differential production and/or maintenance cost of the signal exists [7]. A wealth of investigations have been targeting the evolution of plumage coloration in birds, but the vast majority of these studies focused on the production costs of carotenoid-based and gaudy structural ornamentation (reviews in [8]). In contrast, empirical evidences for such costs in case of melanin-based and depigmented white coloration are scarcer and open to debate, therefore the mechanisms responsible for them are poorly understood [1], [9]–[11].

Plumage ornaments are developed when birds are molting, thus the circumstances prevailing before and during this period are of key importance. In the temperate zone, natural selection favors such an optimal scheduling of main life-history events like breeding and molt, that they are separated [12], [13], since overlapping these energetically conflicting activities induces large costs [14]. Reproduction may affect molting, because (1) low-quality birds are likely to obtain a mate and breed later in the season (e.g. [15]–[18]) and/or (2) a resource-based trade-off may operate between reproduction and molt [19]–[21]. Those birds are more likely to postpone the start of molt which are late breeders [22], which also applies for their late fledging offspring [23], and engage in high investment in current reproduction [24], [25]. This delay leaves a shorter time-window for the plumage to be renewed before the onset of migration or harsh winter weather [13]. Such time constraint is often compensated for by accelerated molt, although this can be costly. For instance, in the European starling *Sturnus vulgaris*, rapid molting compromised the quality of flight feathers [26], while blue tits *Cyanistes caeruleus* with probably reduced time available for molting due to experimentally delayed breeding invested more energy in winter thermoregulation owing to less efficient insulation capacity of the plumage [27]. Molt rate might also negatively affect the elaboration of plumage ornaments, ultimately ensuring signal honesty. Accelerated molt reduced the expression of a carotenoid-based plumage trait in rock sparrows *Petronia petronia* [28], and also the UV/blue coloration in blue tits [29] (‘molt speed constraint’ hypothesis hereafter). However, no studies examined to date whether melanised ornaments are also affected by molt rate, and previous results on pigment-free ornaments [28], [29] do not support the honesty-reinforcing effect of molt rate.

The mechanism behind the effect of molt rate on different pigmented (carotenoid- vs. melanin-based) and structural ornaments (UV/blue vs. depigmented) is probably distinct because of fundamental divergences in how these pigments are incorporated and how structural ornaments are produced, respectively. Carotenoids are transported to and incorporated in growing feathers together with other circulating proteins and lipids [1]. On the contrary, the mechanism for melanin-based coloration mediated by molt rate is probably more direct, given that melanins are manufactured *de novo* in specific cells and organelles (melanocytes and melanosomes, respectively) in the feather follicle from amino acid precursors. For this reason, we hypothesized that if feather production (i.e. keratin synthesis) is accelerated, it could outpace the rate of melanin incorporation or melanogenesis, the latter being often limited by the activity of the enzyme tyrosinase [30], [31], the key enzyme in eumelanogenesis, thus less melanin granules can be embedded in the keratin matrix. As a result smaller and/or duller black signals are expected to develop. UV/blue structural ornaments are composed of large vacuoles, an underlying melanin layer and the medullary spongy layer. The precise arrangement of these components is impeded when molt is accelerated [29]. On the contrary, structural white ornaments always lack the melanin layer, and often the spongy layer too [6], so their appearance depends mostly on the arrangement of the keratin structures and air vacuoles.

The house sparrow *Passer domesticus* is a small passerine, common throughout the world that has been broadly studied with respect to males' plumage ornamentation. The male sparrow possesses a eumelanin-dominated patch of black feathers on their throat and upper breast (bib or, hereafter, ‘badge’) which varies greatly in size among males. Males with larger badge average higher dominance rank according to a recent meta-analysis [32]. Males also possess a depigmented white wing-bar formed by the white tip of the median secondary coverts, the conspicuousness of which also functions in status-signaling [33] or might indicate parasite resistance [34]. The factors that promote honesty to the wing-bar were, however, rarely addressed (see [35] for an exception). We tested the ‘molt speed constraint’ hypothesis, namely that accelerated plumage replacement adversely affects the size and/or coloration of the melanin-based badge and structural white wing-bars of male house sparrows. To this end, we experimentally adjusted molt rate by a photoperiod treatment.

## Materials and Methods

### Ethics statement

Birds were handled in strict accordance with good animal welfare and ethical prescriptions. The protocol for bird care and experimentation adhered to the current Romanian laws and was approved by the Romanian Academy of Sciences (permission #2257).

### Capture and housing

We caught 50 adult male house sparrows by mist nets (Ecotone, Poland) at a cattle farm near Cluj Napoca (46°46′N, 23°33′E, Transylvania, Central Romania) on 25th July 2008. These birds had finished breeding though none had started to molt. Birds were randomly assigned to one of two indoor aviaries (each 4 m L ×3.5 m W ×4 m H, 56 m^3^, *n* = 25 individuals per aviary; see [15] where 45 m^3^ were sufficient for 33 individuals) on the Campus of the Babeş-Bolyai University, Cluj Napoca. Although group housing may potentially confound the results due to inter-male aggression, we housed birds in groups to enable flocking which is characteristic for sparrows year-round and especially in the molting and wintering season [36], instead of individual caging that differs fundamentally from this natural social environment. This setup has also been applied in several previous studies about plumage ornamentation [10], [37]–[40]. However, to exclude that aggression may alter the biological relevance of our results, we tried to moderate aggression by putting two feeding dishes per aviary (see also [40]). Moreover, we registered the aggressive encounters (*n* = 1515) between individually color-ringed birds from a hide through a one-way window using a 10×50 binocular (Zeiss, Germany) during the last month of molt (in average 1 h after the ‘sunrise’ and 1 h before the ‘sunset’ to assess fights for food and roost sites, respectively; totally 1930 min of observations). Only those fights were recorded, which had an unambiguous outcome and both the winner and the loser were identified. From these data we calculated the fighting success (expressed as the proportion of fights won) as a proxy of dominance rank [41] and statistically controlled for this variable by introducing it in models as a covariate (see below). See also the Results section for further justifications that group housing had negligible effects on our results. Although fights were registered only in the last third part of the experiment, this probably represents the social relationships throughout the experiment because dominance rank is known to be consistent at long-term (between seasons) in closed flocks [42].

Each aviary contained bushes (a pile of dead boughs) and nest boxes to provide perch and roost sites (see [43], [44] for more detail on housing). Birds were provided *ad libitum* with high-quality food (protein content ranging between 50–80% [44]), sand and drinking water supplemented with vitamins. To suppress isosporan infection that emerges spontaneously in captive populations, we administered an anticoccidial drug (toltrazuril) to the drinking water [1 mL Baycox 2.5% (Bayer HealthCare, Germany) in 1 L water] for three-day periods on three occasions evenly distributed throughout the experiment. One sparrow from the control photoperiod group (see below) died on 20th August 2008 due to unknown reasons; therefore its data was omitted. Note, however, that the survival rates of wild and other aviary populations are seldom higher [45]. The remaining 49 sparrows were released at their site of capture on 25th November 2008. Some of them were recaptured several months later during the course of other studies conducted on the same population.

### Experimental procedure and data collection

On the day the birds were captured and transferred to the aviaries, the local natural photoperiod was 15L:9D. We started the experimental photoperiod treatment on 28th July 2008 ( = day 1), after 3 days of acclimatization. In one aviary we simulated the natural-like light regime which is characteristic at the latitude of the population by decreasing the daylength in average by 3 min each day (control photoperiod, hereafter ‘CP’ group). In the other aviary we experimentally accelerated the seasonal decrease in photoperiod by decreasing the daylength by 8 min each day (experimental photoperiod, hereafter ‘EP’ group). The photoperiod in this aviary was decreased until it reached 9L:15D (on day 46) and was then held constantly at this level until the end of experiment (day 110). The speed of photoperiod change in EP group, the light intensity and the type of lighting is similar to Serra et al. [28].

We measured wing length (to the nearest 0.5 mm), tarsus length (to the nearest 0.01 mm) and body mass (to the nearest 0.1 g) at capture and release, and mass and molt status (see below) on every 10th day of the 110-day-long experimentation (i.e. 11 measurement sessions). We scored the molt status of each primary feather of both wings following Newton [46]: ‘0’ = old, worn primaries, ‘1’ = dropped primaries, ‘2’, ‘3’ and ‘4’ = one-quarter-, half- and three-quarter-regrown primaries, respectively, and ‘5’ = fully regrown, new primaries. Note that sparrows have 9 primaries, as the 10th (i.e. outermost) is vestigial. Molting index was derived by summing the scores of individual feathers and averaged the scores of the two wings. This score ranges between 0 (all primaries old, molt not started) and 45 (all primaries replaced, molt finished). The molt was considered to have started when the innermost primary was dropped (molt index >0), and to have ended when all primaries had been replaced (molt index = 45). Molt duration refers to the number of days elapsed between the onset and termination of molt. The molt status of the badge and left wing-bar was also recorded as follows: badge, ‘0’ = no molt, ‘1’ = 1–30%, ‘2’ = 31–60%, ‘3’ = 61–99% of feathers growing, respectively, and ‘4’ = all feathers replaced (see [28]); wing-bar, ‘0’ = no molt, ‘1’ = few (1–3), ‘2’ = many (>3) coverts missing, respectively, and ‘3’ = all coverts exchanged.

### Measurement of plumage ornaments

We measured ornament size and brightness from photographs taken before and after molt. We used a Nikon D80 digital camera to photograph each male's badge, holding the head perpendicular to the body, and left wing-bar, with the wing stretched out flat. Photographs were taken in a darkroom using standard lighting conditions with no flash, constant distance from subject and camera settings, and against a metric background (a grid of 1 mm^2^ squares) and black and white standards. Ornament areas were measured in the ImageJ software [47] using ‘set scale’ (with the metric background as template) and ‘freehand selection tool’ by tracing the outline of the ornament. Ornament brightness was measured in the Scion Image software [48] using ‘density slice’ and ‘wand tool’ [33]. We then calculated the relative brightness expressed as a percentage where either the black or the white standard was the 100%, thus the higher values mean ‘blacker’ badge and ‘whiter’ wing-bar. The brightness of these ornaments can be appropriately estimated from photographs since neither has a reflectance peak in the UV-range [49] and there is a strong positive correlation between spectral reflectance values obtained using a spectroradiometer and achromatic brightness measured from photographs [33]. Photographs of 15 randomly chosen sparrows were measured again several months later; repeatability between measures was highly significant for all four ornament variables (all *R*_i_>0.81, all *F*>10.17, all *P*<0.0001).

The feathers composing the badge can be divided into three well separated parts: depigmented white tip, melanised black middle part and plumulaceous basal part. The freshly-molted badge feathers have all three parts, thus the badge of male house sparrows is concealed owing to the white tips which partially cover the neighboring feathers' black middle vanes. These white tips gradually wear off till the next breeding season and the middle black parts, and in turn the badge, become fully exposed. It should be mentioned that the freshly molted, concealed badge area correlates with the fully-expressed, unconcealed badge area in the subsequent breeding season [50]. Moreover, in the present study the visible badge (only the black area) of freshly molted birds measured with Scion Image and the total badge (comprises the black feather parts and white tips, too) measured with ImageJ are significantly positively correlated (Pearson's correlation, *r* = 0.72, *n* = 49, *P*<0.0001). However, since males in different treatment groups may use different tactics to pigment their badge feathers by some concealing more than others with longer white tips, or some growing larger black middle parts than others (Pap PL, Vágási CI, Barta Z unpublished data), to undoubtedly exclude the possibility of faulty badge measurements, we plucked about 5 feathers from the newly-grown badge (from a predetermined region of the breast where the badge is the widest and the badge feathers have the longer white tip). Later, we photographed two randomly chosen feathers against a metric background, measured the length (along the rachis with ImageJ) and area (with Scion Image) of the white tip, black middle part and the total feather, and averaged the values of the two feathers. Afterwards, we computed the proportion of the white tip and black middle part to the total badge feather length and area. All photographs were measured by the same person (CIV) and were performed blind with respect to the treatment.

### Statistical procedures

There were no initial differences between birds placed into different aviaries in biometry, body mass and plumage ornamentation (all *F*<2.08, all *P*>0.16). The molt index of primaries increases following a non-linear S-shaped function, similarly to mass gain during avian ontogeny. Thus, to describe the molting pattern, we fitted a logistic growth curve used for developing nestlings [51] to each bird separately (see also [43], [44]). The logistic function has the form
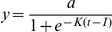
where *y* denotes the molting score at time *t*, *a* is the maximum score or asymptote (i.e. 45 in house sparrows), *K* is the growth constant (indicating molt speed), *I* is the inflection point on the time axis at which molting switches from accelerating to decelerating, while *e* is the base of natural logarithm. We used Mann-Whitney *U*-test to analyze the effect of treatment on the relative day of molt commence. We used general linear models (GLMs) to analyze the effect of photoperiod manipulation on the following response variables: speed of molt (*K* and *I* values), duration of molt, area and brightness of badge and wing-bar. All of these response variables met the assumptions of parametric tests. Treatment was entered as factor, while wing length, tail length, tarsus length, body mass (the mean of 11 measurement sessions; see above), fighting success and pre-molt signal values were all entered as covariates. We built repeated measures ANOVAs to test both between group (photoperiod treatment) and within individual effects. However, to render possible the control for the effects of covariates, we also tested the photoperiod treatment effect on the change in signal values during molt in a GLM by extracting pre-molt from post-molt values [hereafter differential (Δ) signal value]. Because the 4 signal variables may indicate somehow the same abilities, each time we first started with a MANOVA to see whether photoperiod treatment differentiates the two groups by the 4 signal variables. Then, we entered each signal variable separately in GLMs to closely see which variables vary more with photoperiod treatment. In all of these GLM and MANOVA analyses, the non-significant covariate terms were eliminated by a backward stepwise procedure. We present the minimal final models. Mean ± standard error (SE) is shown throughout and all tests were two-tailed with a significance level set at α = 0.05. The sample size was reduced for wing-bar area because 7 birds (3 from CP and 4 from EP group, thus the remaining sample size, *n* = 42, is balanced with 21 individuals in both groups) lost ≥1 coverts before the post-molt photographing.

## Results

### Molt pattern and plumage ornamentation

**Figure 1:**
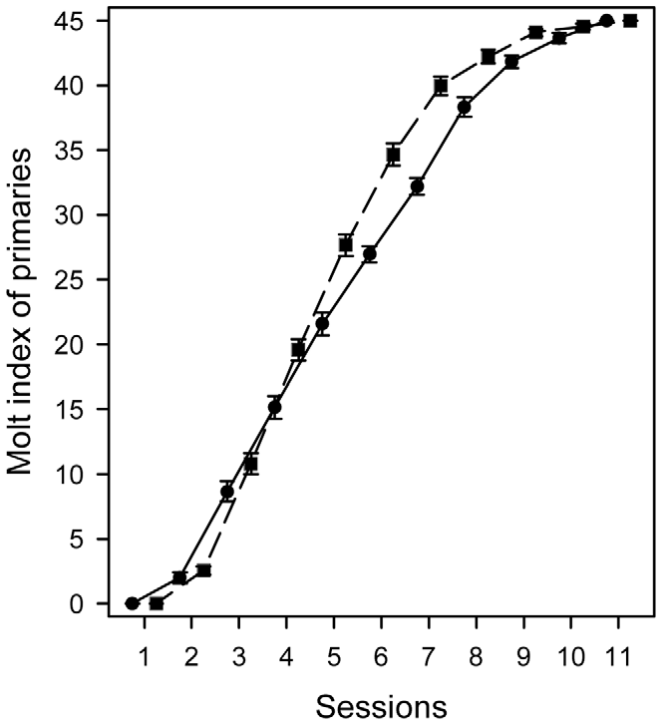
Change in primary molt index during the course of experiment. Male house sparrows from the accelerated ‘EP’ photoperiod group (rectangles and broken line) finished molting two weeks faster than birds in the natural-like ‘CP’ photoperiod group (circles and continuous line). First session is the date of capture (25th July 2008), then each session comes after 10-day intervals. Mean ± SE are shown.

**Figure 2:**
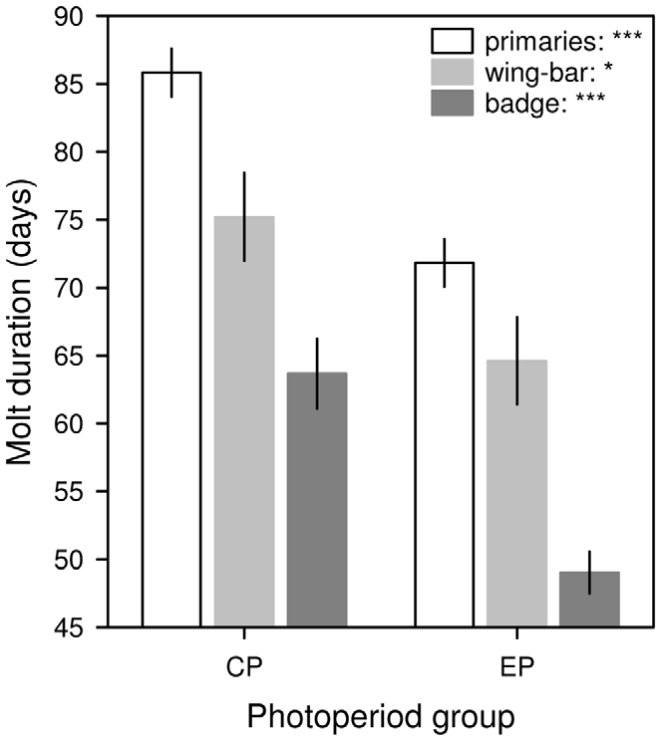
The effect of experimental photoperiod manipulation on molt duration. The duration of replacing the primaries (open columns), wing-bar (light grey columns) and badge feathers (dark grey columns) was significantly shorter if birds were held at experimentally accelerated ‘EP’ photoperiod as related to those at natural-like ‘CP’ photoperiod. Mean ± SE are shown. ‘*’ *P*<0.05, ‘***’ *P*<0.0001.

The median date of molt commence was day 11 for primaries and the wing-bar, and day 24 for the badge. Birds started to molt their primaries and ornamental plumage patches irrespective of treatment (Mann-Whitney *U*-test, *n*_CP_ = 24 and *n*_EP_ = 25 in all cases; primaries: *U* = 271.5, *P* = 0.57; Fig. 1; badge: *U* = 231.0, *P* = 0.17; wing-bar: *U* = 292.5, *P* = 0.88). In contrast, molt duration of EP birds was significantly shorter, roughly by two weeks, compared with conspecifics in the CP group: primaries (mean ± SE in days): CP 85.8±1.8 vs. EP 71.8±1.8 (GLM, *F*_1,40_ = 33.03, *P*<0.0001), badge: CP 63.7±2.6 vs. EP 49.0±1.5 (*F*_1,38_ = 27.78, *P*<0.0001) and wing-bar: CP 75.2±3.2 vs. EP 64.6±3.2 (*F*_1,38_ = 11.07, *P* = 0.0019) (Fig. 2). Analyzing the treatment effect on *K* and *I* values, we found that photoperiod treatment differentiated birds according to molt rate (MANOVA, *F*_2,39_ = 38.95, *P*<0.0001). EP birds molted faster (GLM, *K*-value: *F*_1,40_ = 60.63, *P*<0.0001) and got through the accelerate phase of molt and turned to decelerate phase more rapidly (*I*-value: *F*_1,40_ = 34.25, *P*<0.0001). All the covariates turned out to be non-significant and were eliminated from the final models about molt duration and molt speed.

**Figure 3:**
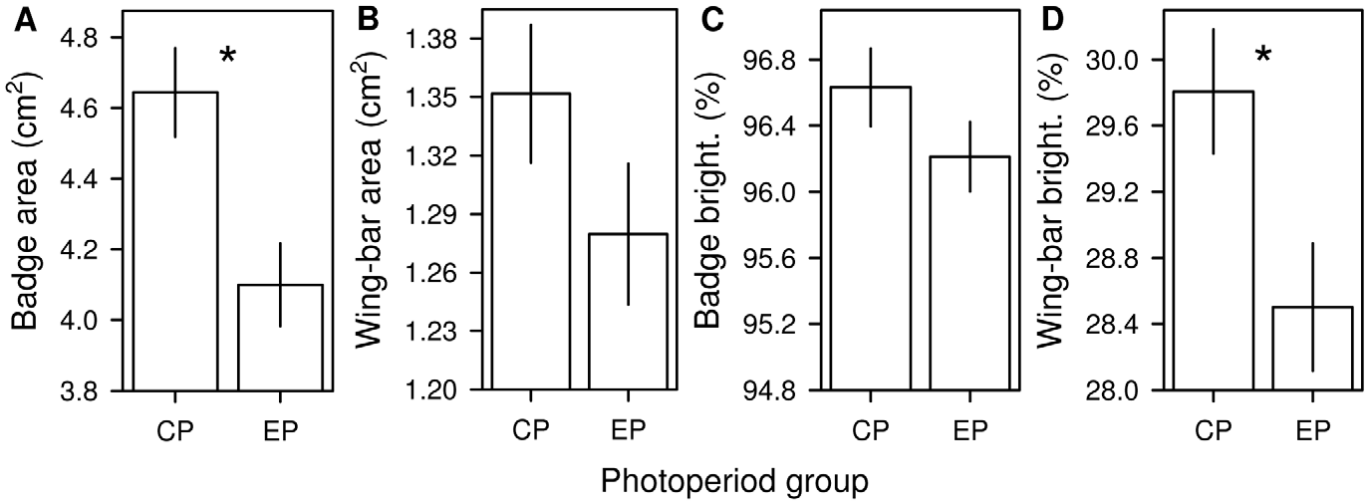
The effect of experimental photoperiod manipulation on plumage ornament expression. Panels show the differences in (A) badge size, (B) wing-bar size, (C) badge brightness and (D) wing-bar brightness between sparrows with experimentally accelerated molting (‘EP’ photoperiod group) and those molting at a natural rate (‘CP’ photoperiod group). Asterisks denote significant differences. Mean ± SE are shown.

Photoperiod treatment groups significantly differed according to the 4 response variables (MANOVA, *F*_4,30_ = 3.55, *P* = 0.017). Table 1 shows the final minimal GLMs about the effect of treatment on each post-molt signal. Sparrows in the EP group that molted faster also developed smaller badges and less bright (i.e. less whitish) wing-bars (Table 1, Fig. 3A, D). However, after Bonferroni correction (α-level/4 response variables = 0.0125) the effect on wing-bar brightness would be non-significant (*P* = 0.038). Although badge brightness and wing-bar area were not significantly influenced by the photoperiod manipulation, EP birds tended to grow less elaborate ornaments in these respects too (Table 1, Fig. 3B, C). Sparrows with longer tarsus had larger-sized wing-bars, and pre-molt badge and wing-bar area positively predicted the post-molt badge and wing-bar area, respectively (Table1). The omission of those 7 individuals from the analyses for which the post-molt wing-bar area cannot be measured (see Statistical procedures) yielded qualitatively similar results regarding the effect of photoperiod on the other signals (GLM with the same minimal models as in Table 1, badge area: *F*_1,36_ = 11.08, *P* = 0.002; badge brightness: *F*_1,36_ = 0.92, *P* = 0.35; wing-bar brightness: *F*_1,37_ = 4.68, *P* = 0.037).

**Table 1:** Final minimal GLMs showing the photoperiod treatment effect on the area and brightness of post-molt plumage ornaments.

Response/source	Estimate	MS	*F*	df	*P*
*Post-molt badge area*					
Intercept	1.69	2.85	13.63	1	0.0006
Pre-molt badge area	0.47	7.39	35.28	1	<0.0001
Photoperiod treatment	0.26	3.00	14.34	1	0.0005
Error		0.21		42	
Final minimal model: *F*_2,42_ = 24.53, *P*<0.0001, *R* ^2^ = 0.54					
*Post-molt wing-bar area*					
Intercept	–0.66	0.02	1.21	1	0.28
Tarsus length	0.08	0.11	7.05	1	0.012
Pre-molt wing-bar area	0.39	0.43	27.52	1	<0.0001
Photoperiod treatment	0.04	0.05	3.16	1	0.084
Error		0.02		35	
Final minimal model: *F*_3,35_ = 11.48, *P*<0.0001, *R* ^2^ = 0.50					
*Post-molt badge brightness*					
Intercept	96.42	445494.3	373042.9	1	<0.0001
Photoperiod treatment	0.21	2.1	1.8	1	0.19
Error		1.2		46	
Final minimal model: *F*_1,46_ = 1.77, *P* = 0.19, *R* ^2^ = 0.04					
*Post-molt wing-bar brightness*					
Intercept	29.28	38547.87	11074.27	1	<0.0001
Photoperiod treatment	0.60	16.04	4.61	1	0.038
Error		3.48		43	
Final minimal model: *F*_1,43_ = 4.61, *P* = 0.038, *R* ^2^ = 0.10					

We found similar results when analyzing the within individual changes in signal values during molt by means of repeated measures ANOVAs. EP birds grew smaller badges (photoperiod treatment [pt]: *F*_1,47_ = 2.43, *P* = 0.13; repeated measures [rm]: *F*_1,47_ = 221.49, *P*<0.0001; pt × rm: *F*_1,47_ = 7.41, *P* = 0.009) and less bright wing-bars (pt: *F*_1,46_ = 0.93, *P* = 0.34; rm: *F*_1,46_ = 805.12, *P*<0.0001; pt × rm: *F*_1,46_ = 6.23, *P* = 0.016) relative to their original pre-molt values as opposed to CP birds. However, after Bonferroni correction (α-level = 0.0125) the effect on wing-bar brightness would turn to marginally significant (*P* = 0.016). Badge brightness (pt: *F*_1,46_ = 2.0, *P* = 0.17, rm: *F*_1,46_ = 32.5, *P*<0.0001, pt × rm: *F*_1,46_ = 0.3, *P* = 0.58) and wing-bar area (pt: *F*_1,37_ = 0.06, *P* = 0.81, rm: *F*_1,37_ = 50.09, *P*<0.0001, pt × rm: *F*_1,37_ = 1.47, *P* = 0.24) were not influenced by treatment. We also found that the Δ signal values significantly differed according to photoperiod treatment (MANOVA, *F*_4,32_ = 3.01, *P* = 0.03). We then analyzed each signal's Δ value separately. However, since in none of the analyses did any significant covariate term remain, these models yielded the same results as the repeated measures ANOVAs (not shown).

### Condition, fights and pigmentation pattern of badge feathers

Treatment had non-significant effect on body mass measured at 10-day intervals during the whole experiment (repeated measures ANOVA, *F*_1,47_ = 0.02, *P* = 0.90) and during the period when the aggressive encounters were observed (*F*_1,47_ = 0.11, *P* = 0.74). Similarly, treatment did not affect the number of fights in which an individual was involved (GLM, *F*_1,27_ = 2.51, *P* = 0.13) and the number of fights initiated by the individual (*F*_1,27_ = 0.35, *P* = 0.56) per hour of observation. These results demonstrate that birds in the EP group were not more stressed or aggressive due to the perceived accelerated commence of winter than their CP counterparts. Interestingly, birds with larger pre- and post-molt wing-bar area were involved in fewer fights (pre-molt wing-bar area: *F*_1,27_ = 4.61, *P* = 0.04; post-molt wing-bar area: *F*_1,27_ = 14.21, *P* = 0.0008), and those with larger post-molt wing-bar also initiated fewer fights (*F*_1,27_ = 6.84, *P* = 0.015).

We did not find treatment effect either on absolute length or on absolute area of the white tip and black middle part of badge feathers. When the proportion-to-total badge feather length and area (i.e. relative length and area) was considered, we found similar non-significant effects for both badge feather parts (all *F*<3.05, all *P*>0.09).

## Discussion

The ‘molt speed constraint’ hypothesis was largely supported for melanin-based and depigmented plumage ornaments. We have experimentally demonstrated that individuals which completed their molt faster by roughly two weeks paid a cost in terms of plumage ornament expression, because molt rate adversely affected the badge size and the wing-bar brightness. The effect on badge size might be a by-product if EP birds conceal the ‘visible badge’ more by the buff white feather tips, but the facultatively exposable ‘hidden badge’ (sensu [52]; see also Materials and Methods) did not differ between groups. However, this can be rejected as the length, area and the proportion in length and area of the feather that was comprised of the white tip were similar between treatment groups. These, together with the fact that the black middle parts' absolute or proportional length and area was also not influenced, advocate that the number of melanised badge feathers was affected similarly to the effect of a diet treatment in zebra finches *Taeniopygia guttata* [53].

Studies of melanin-based signaling have centered mostly on ultimate functions rather than proximate mechanisms that ensure reliability [53]. Empirical evidence suggests the existence of maintenance (e.g. [52], [54] and production costs as well, but the mechanistic basis of the latter remained unclear. To discuss in detail the several hypotheses that have been put forward to find production costs for melanised plumage traits is beyond the scope of this study (but see thorough reviews in [1], [11]). Briefly, some studies have focused on the nutritional condition during molt. Juvenile male house sparrows with higher blood protein levels grew larger badges [37] and those supplemented with an essential precursor of melanin, the phenylalanine, grew blacker badges [35], while zebra finches fed with extra calcium, which is cofactor of melanin-producing enzymes, grew larger black signals [53; but see 55]. Galván and Alonso-Alvarez [56] demonstrated the inhibitory effect of an antioxidant, the glutathione, on the melanised breast stripe of great tits *Parus major*. Others studied the effect of circulating hormone titers. Buchanan et al. [57] showed that in male house sparrows, testosterone implants enhanced both the metabolic rate and badge size. Roulin et al. [58] evidenced that corticosterone-implanted juvenile barn owls *Tyto alba* developed plumage with less phaeomelanin. All of these factors can rely on condition-dependence, as presumably prime quality birds have better access to food resources, endure more the elevated metabolic costs of testosterone-dependent ornament production, and have lower stress-induced corticosterone profiles. However, other studies could not detect nutritional influence (e.g. [38]), while the link between immunocompetence and coloration mediated by androgen hormones was partially supported and is more complicated than previously thought (e.g. [39]).

Here, we experimentally tested the ‘molt speed constraint’ hypothesis as a proximate mechanism that might ensure the honesty of eumelanin-based ornaments. There are several reasons to believe that variation in molt speed could explain variation in badge size and/or coloration in house sparrows. First, the two weeks difference in molt duration between our control and experimental birds was well within the range of variation that naturally occurs within most wild passerines, including house sparrows [36]. Second, we found an association between molt duration and badge size even though our birds were provided with high-quality food *ad libitum*, therefore we expect a stronger effect under field conditions where resources are more limited. Third, it requires a rate-limiting factor in the biochemical pathway of endogenous melanin synthesis, a prerequisite which may be fulfilled by the activity of the enzyme tyrosinase [30], [31] that catalyzes several biochemical reactions in the whole course of eumelanin synthesis [1]. If the process of eumelanin synthesis is of lower efficiency (e.g. reduced tyrosinase activity), a smaller and/or less intense black ornament is expected to develop [59]. However, the lower rate of melanin production may also lead to similarly sized but duller black badges (i.e. fewer pigment granules deposited per feather), but this was not the case in our experimental photoperiod group. This means that instead of a tyrosinase rate limitation there is a threshold effect in the distribution of active, pigment-producing melanosomes. Those feathers that are positioned at the bottom periphery of the badge had melanosomes activated at all in the faster molting group, thus fewer badge feathers were colored to black but with similar intensity (see also [53]). It remains for future studies to shed more light on the cellular or molecular bases of melanin granule distribution on and among feathers to elucidate why the number of melanised feathers is affected instead of the coloration [53].

Depigmented white signals are largely unexplored in terms of whether they also have production costs. Since these plumage traits do not contain any pigment and are thus emancipated from the production costs ascribed to melanins or carotenoids, they are presumed to incur mostly maintenance costs such as an increased risk of predation [60] or social control from conspecifics [61]. However, contrary to the previously established ‘cheap-to-produce’ concept, empirical evidence is accumulating that the expression of depigmented plumage is also costly (e.g. [10], [34], [62]–[64], Pap PL, Vágási CI, Barta Z unpublished data; but see [65]). However, the mechanism responsible for these apparent production costs is unclear. White structural signals are produced by randomly distributed and differently sized air-filled vacuoles embedded in the feather keratin that lacks the underlying melanin layer [6], [66]. The magnitude of light reflected by white plumage patches depends on the interface between feather keratin and air-filled vacuoles, such that feathers that incorporate larger vacuoles into their structure appear brighter [6]. A medullary spongy layer may also be present since white plumage ornaments have evolved from structural blue feathers as a result of derived loss of the underlying melanin deposition (at least in manakins *Lepidothrix* spp. [6]; see also [66] for a case of amelanism). The precision of the arrangement of this nanometer-scale structure may also contribute to variation in the color produced [67]. Although the microscopic analysis of the nanometer-scale structure of house sparrows' white wing-bar-forming coverts are missing to date, as far as we are aware, it is possible that the birds which had undergone a rapid molt were unable to (1) either impute large vacuoles and/or (2) precisely structure the spongy layer in the ways required to produce a bright white feather patch. This scenario suggests that the appearance of white feathers is not limited by any particular resource-demand but by the ability to develop feathers with the correct microstructure, ensuring ultimately its honesty [64]. To the best of our knowledge, this study is the first to ascertain a mechanism that mediates the production cost of a depigmented, non-UV structural signal. The treatment effect on wing-bar brightness should be treated with circumspection, since after Bonferroni correction it disappeared. Albeit, we consider this result important because the treatment × repeated measures remains marginally significant and this correction procedure was recently severely criticized (e.g. [68]). Previous studies that addressed these questions did not found any effect of molt speed on the size [28], [29] or brightness [29] of white signals. Regarding coloration, the microscopic analysis of feather barbs/barbules is indispensable to disentangle whether this discrepancy is due to species (blue tits vs. house sparrows) differences in feather microstructure.

This study did not test explicitly a condition-dependent model of ornamentation, however, we can make speculative inferences on how honest signaling may be guaranteed by molt rate. The timing of molt may be connected to condition in two non-exclusive ways. Lower quality males often acquire a mate later in the season (e.g. [15]–[17]), thus early breeders are usually in better condition [18], [29]. Late breeders may have lower survival prospects [46] further indicating their lower quality. Alternatively, birds that undertake high parental investments in current reproduction relative to their condition may run out of time and/or deplete their finite resources [69], reflecting a trade-off between breeding and molt [19]–[21]. For instance, Griffith [70] found that male house sparrows with experimentally increased breeding effort expressed smaller-sized badges in the subsequent molt. Moreover, juveniles males which left the nest relatively late in the season also grew smaller badges [71], [72]. Thus, birds of inferior condition may initiate their molt later and then they cannot bluff their abilities because of faster molting. Only birds of superior condition that have a relaxed molt might be able to incorporate more melanins in their feathers and arrange the nanostructure precisely. Since the photoperiod treatment affected all birds in a similar way, our results are more consonant with the latter explanation. Notwithstanding, future experiments that jointly manipulate photoperiod and condition or hormonal profiles (e.g. corticosterone [58], prolactin [69]) linked to condition are encouraged to gain a clearer picture. Our findings emphasize that, through a cascade effect (delayed breeding is followed by delayed molt), molt couples the pre- and post-molt events. Molt speed may mediate the hidden costs of late breeding and/or high parental activity [69] by diminishing plumage ornamentation. Given that both the melanin-based badge and the depigmented wing-bar are subjects to intra- and intersexual selection [15], [32], [33], [73], the coupling of successive breeding attempts by molt could explain the strong relationship between the condition-dependent ornamentation and annual [15] or lifetime reproductive success [74]. We show that the time available for molt, and thus the photoperiod it was exposed to, is an environmental factor that can generate variation in melanin-based and depigmented plumage traits (see [8], [75] and references therein). This also highlights the selective advantage attained by birds which breed and fledge their nestlings earlier in the season [28], [29].
